# Autistic-Like Behaviors, Oxidative Stress Status, and Histopathological Changes in Cerebellum of Valproic Acid Rat Model of Autism Are Improved by the Combined Extract of Purple Rice and Silkworm Pupae

**DOI:** 10.1155/2016/3206561

**Published:** 2016-01-04

**Authors:** Nartnutda Morakotsriwan, Jintanaporn Wattanathorn, Woranan Kirisattayakul, Kowit Chaisiwamongkol

**Affiliations:** ^1^Integrative Complementary Alternative Medicine Research and Development Center, Khon Kaen University, Khon Kaen 40002, Thailand; ^2^Department of Anatomy and Graduate School, Faculty of Medicine, Khon Kaen University, Khon Kaen 40002, Thailand; ^3^Department of Physiology, Faculty of Medicine, Khon Kaen University, Khon Kaen 40002, Thailand; ^4^Department of Anatomy, Faculty of Medicine, Khon Kaen University, Khon Kaen 40002, Thailand

## Abstract

Due to the crucial role of oxidative stress on the pathophysiology of autism and the concept of synergistic effect, the benefit of the combined extract of purple rice and silkworm pupae (AP1) for autism disorder was the focus. Therefore, we aimed to determine the effect of AP1 on autistic-like behaviors, oxidative stress status, and histopathological change of cerebellum in valproic acid (VPA) rat model of autism. VPA was injected on postnatal day (PND) 14 and the animals were orally given AP1 at doses of 50, 100, and 200 mg·kg^−1^ BW between PND 14 and PND 40. The autism-like behaviors were analyzed via hot-plate, rotarod, elevated plus-maze, learning, memory, and social behavior tests. Oxidative stress and the histological change in the cerebellum were assessed at the end of study. AP1 treated rats improved behaviors in all tests except that in hot-plate test. The improvement of oxidative stress and Purkinje cell loss was also observed in the cerebellum of VPA-treated rats. Our data suggest that AP1 partially reduced autism-like behaviors by improving oxidative stress and Purkinje cell loss. Further research is required to identify the active ingredients in AP1 and gender difference effect.

## 1. Introduction

The prevalence of autism, a pervasive neurodevelopment disorder, is dramatically increasing, over the past decade [1]. It is characterized by impaired social interactions, language deficit, and stereotype behavior [2]. It has been reported that around 70% of people with autism exhibit cognitive deficit [3]. Due to these impairments, the quality of life of both the autism patients and the caregivers is disturbed. In addition, autism also produces a great burden on socioeconomic costs [4]. Therefore, the effective intervention which is safe, cheap, and easy to access is essentially required. Unfortunately, no current treatment can completely cure this disorder.

Recently, substantial evidence has demonstrated that cerebellum dysfunction plays a pivotal role in the pathogenesis of autism. Autism patients show numerous changes in the cerebellum, including Purkinje cell loss [5], oxidative stress elevation [6], and GABAergic system dysfunction [7]. These changes in the cerebellum are reported to play a pivotal role on the pathophysiology of core symptoms of autism such as motor, memory, and language impairment [8].

Growing evidence has shown that substances possessing antioxidant [9] and neuroprotective activities, such as* Bacopa monnieri* [10, 11], resveratrol [12], green tea [13], and piperine [14], can also mitigate autism-like behavior in the valproic acid (VPA) rat model of autism [15–18]. Therefore, we hypothesized that a substance possessing antioxidant and neuroprotective effects might provide benefits for patients with autism.

Purple rice or* Oryza sativa* (purple color) and pupae of silkworm or* Bombyx mori* are widely consumed in the Northeast region of Thailand. Our previous works have clearly shown that both purple rice extract and silkworm pupae extract exhibit antioxidant and neuroprotective effects [19, 20]. Based on this information, the effect of the combined extract of purple rice and silkworm pupae (AP1) on autistic-like behaviors and the possible underlying mechanisms in VPA-autistic rat model were investigated.

## 2. Materials and Methods

### 2.1. Preparation of “AP1,” the Combined Extract of Purple Rice and Silkworm Pupae

The purple rice or* Oryza sativa* (purple color) used in this study was harvested in December 2011 from Khon Kaen Province. The rice was prepared as a water extract by using the decoction method. Then, the extract was filtered and centrifuged at 2500 rpm for 10 minutes. The supernatant was kept at −40°C for 30 minutes and evaporated using a lyophilizer. The yield was 3.75%.

The pupae of* Bombyx mori* or silkworm used in this study were male Thai native silkworm pupae var. Nangnoi. They were collected from May to July 2011 from Queen Sirikit Sericulture Center, Khon Kaen, Thailand. The silkworm pupae were prepared as an ethanolic extract (95%) by using the percolation technique. The yield of the obtained extract was 9.82%.

The extract of purple rice and the extract of silkworm pupae were mixed at a ratio of 6 : 1 (w/w), which provided the highest antioxidant effect via 2,2-diphenylpicrylhydrazyl (DPPH) and ferric reducing activity of plasma (FRAP) assays and showed more benefit than either purple rice extract or silkworm pupae alone. The total phenol content in AP1 was 312.41 ± 12.04 mg of gallic acid equivalent (GAE)/100 g of plant, whereas the anthocyanin content was 298.16 ± 11.43 mg/L cyaniding-3-glucoside equivalent/mg extract. The main amino acids in AP1 were lysine and tyrosine, and the contents of various amino acids in AP1 are shown in Table 1.

### 2.2. Animals and Treatment

Pregnant rats were purchased from National Laboratory Animal Center, Salaya, Nakorn Pathom. Both male and female offspring rats at the age of 14 days were used as experimental animals. The weights of the rat pups on the first day of experiment were 20–40 grams. All animals were randomly housed 6 per cage with a 12 h-12 h light-dark cycle and were given access to food and water ad libitum. Sodium salt of valproic acid (VPA) (Sigma-Aldrich) in 0.9% saline (100 mg/mL, pH 7.3) at dose of 400 mg·kg^−1^ was freshly prepared and administered to the rat pups on postnatal day 14 (PND 14) via a subcutaneous route [21, 22]. The experimental protocols had been approved by the Institutional Animal Care and Use Committee, Khon Kaen University, Thailand (Record number AEKKU 13/2556, Ref. number 0514.1.12.2/110).

### 2.3. Experimental Protocol

The experiment was performed in both male and female offspring. Each group contained the same amount of male and female offspring (5 males and 5 females, per group). They were divided into various groups as follows. 

*Group I.* Naïve control: the offspring rats in this group received no treatment. 

*Group II.* VPA group: the animals in this group were administered valproic acid (VPA) alone. 

*Group III.* VPA + vehicle: all animals in this group were administered VPA and were treated with a vehicle.

*Groups IV–VI*. VPA + AP: the offspring rats in these groups were treated with AP1 at doses of 50, 100, and 200 mg·kg^−1^ of body weight (BW), respectively.

Only the offspring rats which showed autism-like behaviors were selected for the study and were assigned to groups II–VI. The assigned substances were administered once daily from PND 14 to PND 40. In addition, all behaviors of experimental animals were determined via a battery test consisting of negative geotaxis, hot-plate, rotarod, open-field, elevated plus-maze, Morris water maze, and social behavior tests. On PND 41, the cerebellum of each rat was isolated, and the oxidative stress markers, including the level of malondialdehyde (MDA) and the activities of superoxide dismutase (SOD), catalase (CAT), and glutathione peroxidase (GSH-Px) enzymes, as well as the density of Purkinje cells in the cerebellum were measured.

### 2.4. Determination of Behavior Disorders 

#### 2.4.1. Negative Geotaxis

The experimental rats were determined negative geotaxis by placing them on a 45° slope with its head pointing down the decline. The duration time to turn 180° was recorded. This test was performed on PND 14–19 [16].

#### 2.4.2. Mid-Air Righting Reflex

Mid-air righting reflex was performed on PND 17–19 by dropping the offspring rats upside down from a height onto a padded surface. The time duration of rostrocaudal movements and the ability to land by their hind limbs were observed and recorded as an index [16].

#### 2.4.3. Hot-Plate Test

Decreased pain sensitivity is one of the common features of children with autism [23], so the pain response to thermal stimuli was evaluated between PND 37 and PND 39 by using a plantar test apparatus (Ugo Basile, Comerio, Italy) according to Hargreaves' method [24]. The withdrawal response latency was recorded and used as indicator of pain response to thermal stimuli.

#### 2.4.4. Social Interaction

Based on the finding that the impairment of social interaction is an important clinical manifestation in autism [25], the effect of AP1 on social behavior was also determined. In order to enhance social interactions, the animals were separated and housed individually the night before the experiment.

They were matched for their gender and weight. Two animals from the same group, but different litters and cages, were placed into an acrylic plastic circular cage under red light for 20 min on PND 40. Pairs were tested in a randomized order for groups, and the paired rats did not have a difference in body weight of more than 15 g. The frequency of the following parameters was used as indicators of social engagement [26]: pining, following, grooming each other, sniffing body parts other than anogenital body parts, and sniffing anogenital body parts.

#### 2.4.5. Elevated Plus-Maze Test

Although anxiety is not the main clinical manifestation of autism, anxiety is a real difficulty for patients with autism [27]. Therefore, we also evaluated anxiety by using the elevated plus-maze test. All animals were directly placed on the elevated plus maze consisting of two open and two closed arms (50 cm length × 12 cm wide × 30 cm high) and placed approximately 50 cm from the floor. Each rat was placed in the center of the maze facing an open arm and was allowed to freely explore for 5 min. The percent of time spent in the open arm and the percent of time spent in entering the open arm entries were recorded.

#### 2.4.6. Morris Water Maze Test

The effect of AP1 on memory was evaluated based on the previous finding that autism patients often display a memory deficit [28]. The animals were exposed to a pool consisting of 4 quadrants (Northeast, Southeast, Southwest, and Northwest) which was filled with tap water (25°C, 40 cm deep) and covered with nontoxic milk. The removable platform was immersed below the water surface of one quadrant. Each animal had to memorize the location of the immersed platform by using specific visual cues placed around the outside of the tank. The time for each rat spent to find and climb on the immersed platform was recorded as the escape latency. Twenty-four hours later, the animal was reexposed to the same condition, except that the immersed platform had been removed. The mean time that each rat spent in the target quadrant in order to search for the missing platform was noted as the retention time and was used as the index of retrieval memory. Both the escape latency and the retention time in a 5-minute exposure time were used as indices of learning and memory. A blind observer always stood at the same position, and care was taken not to disturb the relative location of water maze with respect to other objects in the laboratory. The determination of the spatial memory capacity via this test was done on PND 40 [29].

#### 2.4.7. Rotarod Test

Each animal was placed on a rod rotating at a speed of 40 revolutions per minute (rpm) on PND 24 to PND 26. The time taken by each animal to maintain its balance on the rod within a 5-minute period was recorded as the endurance time [29].

#### 2.4.8. Stereotype Behaviors

The effect of AP1 on stereotype pattern behaviors such as rearing, licking, and grooming was assessed on PND 40. The open-field test is widely used to measure anxiety-related behavior in addition to general locomotor and explorative activity. The open field consisted of a square arena with a diameter of 90 cm and surrounded by a wall of 70 cm height. At the start of the test, the rat was transported to the arena in a clean and empty cage. The cage was covered by a perspex lid. The cage was turned and put in the center of the arena. The lid at the bottom was carefully removed and the cage was lifted, allowing the rat to explore the arena for 5 min. Horizontal and vertical locomotive activity were measured for 5 min in an open field including stereotyped behaviors such as rearing, licking, and grooming which were also measured. The open field was cleaned thoroughly before each test [30].

### 2.5. Determination of Oxidative Stress Markers

At the end of study, the cerebellum was isolated and was prepared as a homogenate by using 0.1-M phosphate buffer (pH 7.4). Then, a homogenate was centrifuged at 12,000 rpm at 4°C for 20 minutes. The supernatant was used to determine the oxidative stress markers, including the level of malondialdehyde (MDA) and the activity of superoxide dismutase (SOD), glutathione peroxidase (GSH-Px), and catalase (CAT), by using methods explained elsewhere. The MDA level was determined via the thiobarbituric acid reaction [31]. The activity of SOD was determined by measuring the rate of reduction of cytochrome c at 550 nm [32]. The CAT activity in the supernatant was determined by monitoring the rate of decrease of H_2_O_2_ at 240 nm [33], whereas GSH-Px activity was determined by measuring the rate of decrease of nicotinamide adenine dinucleotide phosphate (NADPH) at 340 nm [34]. All data were expressed as units per milligram protein.

### 2.6. Histomorphology Study

On PND 40, animals were anesthetized with sodium thiopental at a dose of 60 mg·kg^−1^ BW via intraperitoneal injection and were perfused with 4% paraformaldehyde in 0.1-M sodium phosphate buffer (pH 7.4). The cerebellum of each rat was removed and postfixed in 30% sucrose in phosphate buffer overnight. Serial sections of the brains were cut to a thickness of 20 *μ*m via a microtome in a cryostat. Sections were stored in phosphate buffer, and they were placed on slides coated with a 0.01% aqueous solution of high-molecular-weight poly-L-lysine solution. Then, they were stained with hematoxylin and eosin.

### 2.7. Statistical Analysis

Data were presented as mean ± standard error of mean (SEM). Statistical significance was analyzed by using the one-way analysis of variance (ANOVA), followed by the Tukey HSD post hoc test. Probability levels less than 0.05 were accepted as significance.

## 3. Results

### 3.1. Effect of AP1 on Negative Geotaxis

**Figure 1:**
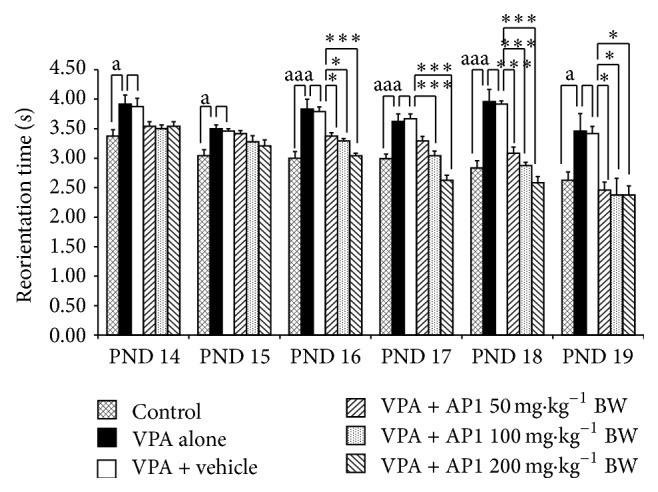
Effect of AP1 on negative geotaxis of behavior. Data were presented as mean ± SEM. ^a^ *p* value < 0.05 and ^aa^ *p* value < 0.005, compared to the control group. ^*∗*^ *p* value < 0.05 and ^*∗∗∗*^ *p* value < 0.001, compared to the VPA + vehicle group.

The data for the effect of AP1 on the negative geotaxis behavior are shown in Figure 1. The effect of AP1 on negative geotaxis behavior was assessed between PND 14 and PND 19. Our data showed that VPA-treated rats significantly increased latency to reorient themselves on the negative geotaxis test from PND 14 to PND 19 (*p* value < 0.05, 0.05, 0.001, 0.001, 0.001, and 0.05, resp., compared to control rats). When compared to the rats treated with the vehicle alone, no significant change of this parameter was observed in the rats treated with VPA + vehicle. VPA-treated rats that received AP1 at a dose of 50 mg·kg^−1^ BW significantly decreased reorientation latency on PND 16, PND 18, and PND 19 (*p* value < 0.05, 0.001, and 0.05, resp., compared to rats treated with VPA + vehicle). AP1 at doses of 100 and 200 mg·kg^−1^ BW reduced the elevated reorientation latency in VPA-treated rats on PND 16, PND 17, PND 18, and PND 19 (*p* value < 0.05 and 0.001, 0.001 all, 0.001 all, and 0.05 all, resp., compared to rats treated with VPA + vehicle).

### 3.2. Effect of AP1 on Mid-Air Righting Reflex

**Figure 2:**
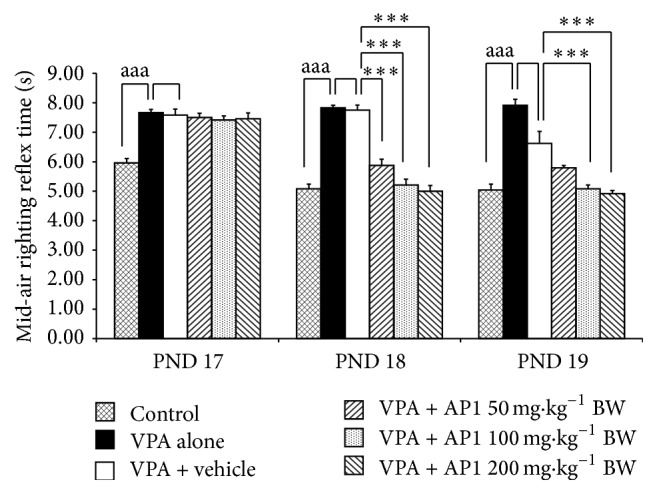
Effect of AP1 on the mid-air righting reflex. Data are presented as mean ± SEM. ^aaa^ *p* value < 0.001 compared to the control group and ^*∗∗∗*^ *p* value < 0.001, compared to the VPA-treated group that received the vehicle.

Figure 2 shows that VPA-treated rats demonstrated the significant increase in the mid-air righting reflex time on PND 17–PND 19 (*p* value < 0.001 all, compared to control rats). The vehicle failed to produce significant changes of this parameter in the VPA-treated rats. The medium and the high doses of AP1 decreased the elevation of mid-air righting reflex time on PND 18 and PND 19 (*p* value < 0.001 all, compared to the rats that received VPA + vehicle), whereas the low dose of AP1 produced the significant reduction of the mid-air righting reflex time only on PND 19 (*p* value < 0.001, compared to the rats that received VPA + vehicle).

### 3.3. Effect of AP1 on Pain Induced by Thermal Stimuli

**Figure 3:**
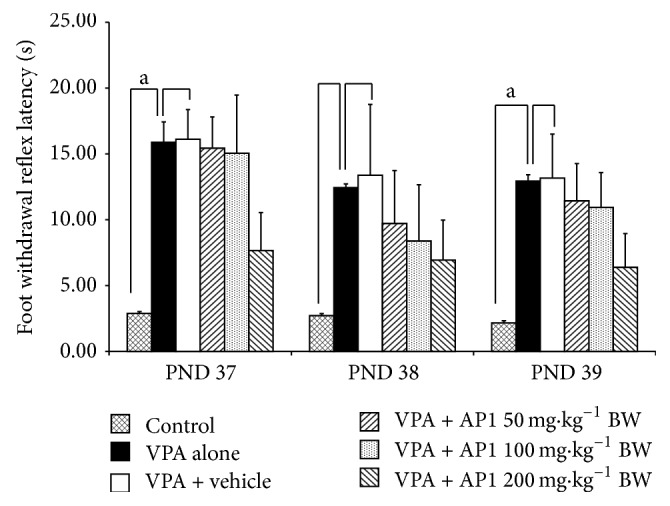
Effect of AP1 on pain induced by thermal stimuli, as evaluated by using the hot-plate test. Data are presented as mean ± SEM. ^a^ *p* value < 0.05, compared to the control group.

The effect of AP1 on thermal nociception was shown in Figure 3. VPA-treated rats exhibited significantly increased foot withdrawal reflex latency on PND 37 and PND 39 (*p* value < 0.05 all, compared to the control rats). Neither the vehicle nor any dose of AP1 was found to produce a significant change in the foot withdrawal reflex latency in VPA-treated rats.

### 3.4. Effect of AP1 on Social Interaction

**Figure 4:**
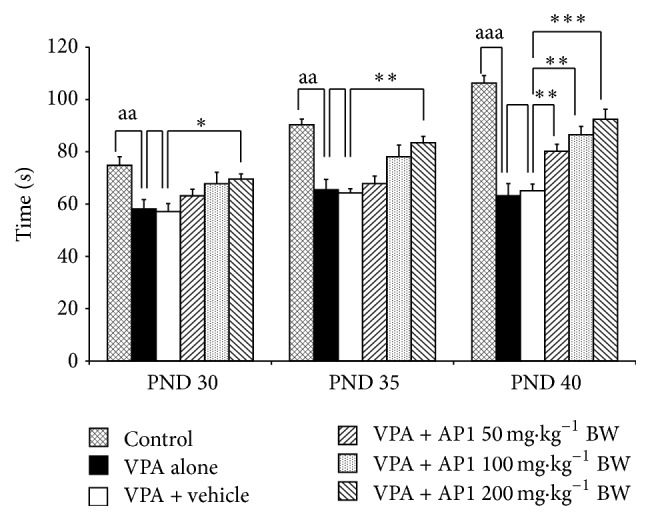
Effect of AP1 on social behavior. Data are presented as mean ± SEM. ^aa^ *p* value < 0.005, ^aaa^ *p* value < 0.001, compared to the control group. ^*∗*^ *p* value < 0.05; ^*∗∗*^ *p* value < 0.005; and ^*∗∗∗*^ *p* value < 0.001, compared to the VPA-treated group that received the vehicle.

Figure 4 showed that rats which received VPA significantly decreased social behaviors at PND 30, PND 35, and PND 40 (*p* value < 0.005, 0.005, and 0.001, resp., compared to control group). VPA rats which received AP1 at dose of 200 mg·kg^−1^ BW significantly enhanced social behaviors throughout the study period (*p* value < 0.05, 0.005, and 0.001, resp., compared to VPA rats which received vehicle), while VPA rats which received AP1 at doses of 50 and 100 mg·kg^−1^ BW showed the increased social behaviors only at PND 40 (*p* value < 0.005 all, compared to VPA rats which received vehicle).

### 3.5. Effect of AP1 on Anxiety

**Figure 5:**
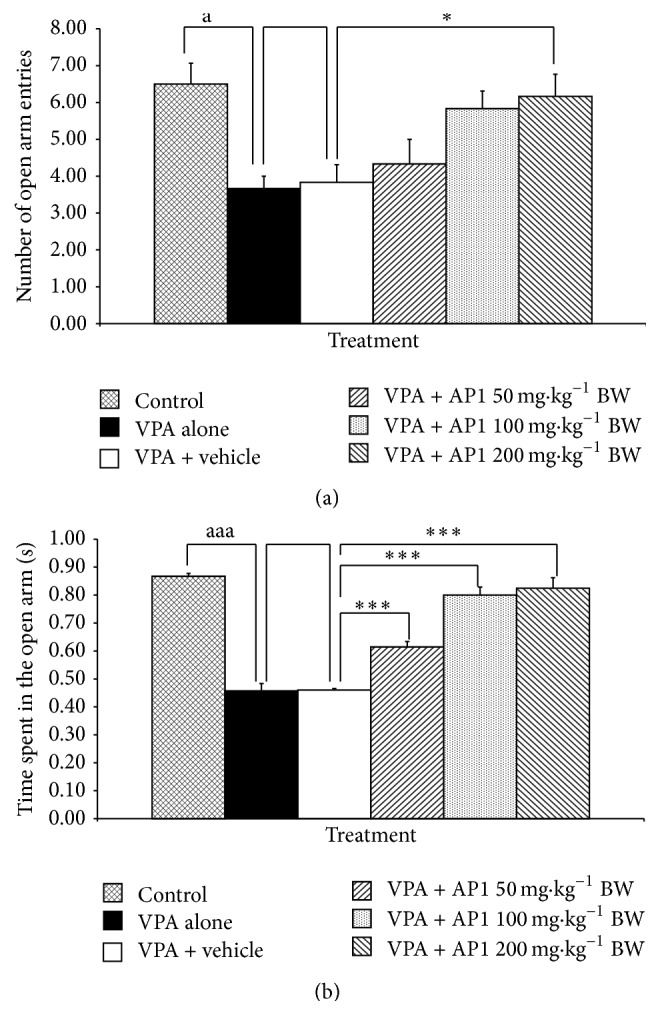
Effect of AP1 on anxiety evaluated by using the elevated plus-maze test: (a) effect of AP1 on the number of open arm entries and (b) effect of AP1 on the time spent in the open arm. Data are presented as mean ± SEM. ^a^ *p* value < 0.05 and ^aaa^ *p* value < 0.001, compared to the control group. ^*∗*^ *p* value < 0.05, compared to the VPA-treated group that received the vehicle.

The effect of AP1 on anxiety was evaluated by using the elevated plus-maze test, and the results were shown in Figures 5(a)-5(b). VPA significantly decreased the number of open arm entries and the time spent in the open arm (*p* value < 0.05 and 0.001, resp., compared to the control rats). The vehicle failed to produce any significant changes in this parameter. AP1 at a dose of 200 mg·kg^−1^ BW significantly increased the number of open arm entries and the time spent in the open arm in VPA-treated rats (*p* value < 0.05 and 0.001, resp., compared to the VPA-treated rats that received the vehicle). However, the low and the medium doses of AP1 only produced significant increases in the time spent in the open arm (*p* value < 0.001 all, compared to the VPA-treated rats that received the vehicle).

### 3.6. Effect of AP1 on Memory

**Figure 6:**
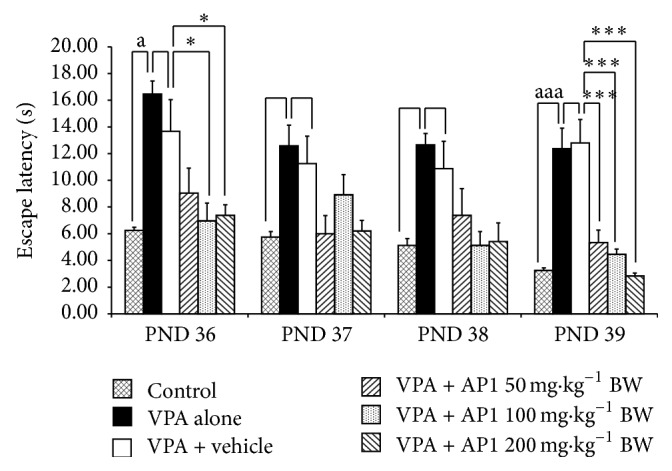
Effect of AP1 on escape latency in the Morris water maze test. Data are presented as mean ± SEM. ^a^ *p* value < 0.05 and ^aaa^ *p* value < 0.001, compared to the control group. ^*∗*^ *p* value < 0.05 and ^*∗∗∗*^ *p* value < 0.001, compared to the VPA-treated group that received the vehicle.

Figure 6 showed the effect of AP1 on the escape latency on the Morris water maze test. The escape latency of VPA-treated rats was significantly increased on PND 36 and PND 39 (*p* value < 0.05 and 0.001, resp., compared to the control rats). VPA-treated rats that also received the vehicle showed no significant changes in this parameter when compared to the rats treated with the vehicle treatment. VPA-treated rats that received AP1 at doses of 100 and 200 mg·kg^−1^ BW showed significant attenuations in the enhanced escape latency time on PND 36 and PND 39 (*p* values < 0.05 all and 0.001 all, compared to the VPA-treated rats that received the vehicle). However, AP1 at a dose of 50 mg·kg^−1^ BW produced a significant attenuation in the enhanced escape latency only on PND 39 (*p* value < 0.001, compared to the VPA rats that received the vehicle).

### 3.7. Effect of AP1 on Motor Coordination

**Figure 7:**
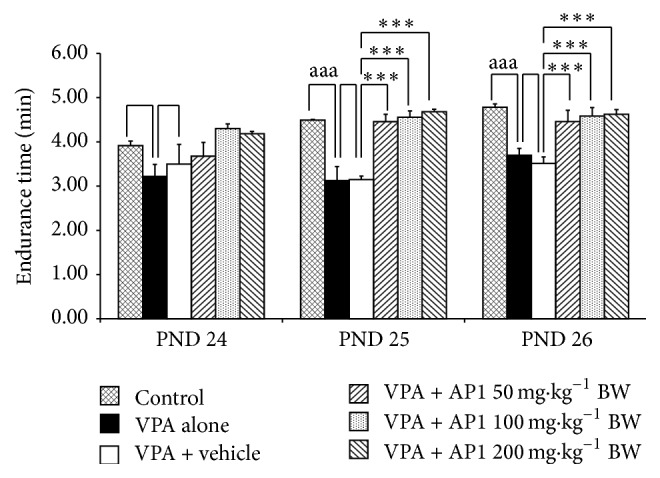
Effect of AP1 on sensory-motor coordination in rotarod test. Data are presented as mean ± SEM. ^aaa^ *p* value < 0.001, compared to the control group. ^*∗∗*^ *p* value < 0.005 and ^*∗∗∗*^ *p* value < 0.001, compared to the VPA-treated group that received the vehicle.

The effect of AP1 on motor coordination was assessed via the rotarod test during the period from PND 24 to PND 26, and the results were shown in Figure 7. The results revealed that the VPA-treated rats showed a significant decrease in endurance time on PND 25 and PND 26 (*p* value < 0.001 all, compared to the control rats). The vehicle was found not to produce any change on this parameter in the VPA-treated rats. However, all doses of AP1 did mitigate the changes induced by VPA (*p* value < 0.001 all, compared to the VPA-treated rats) that also received the vehicle.

### 3.8. Effect of AP1 on Stereotype Behaviors

**Figure 8:**
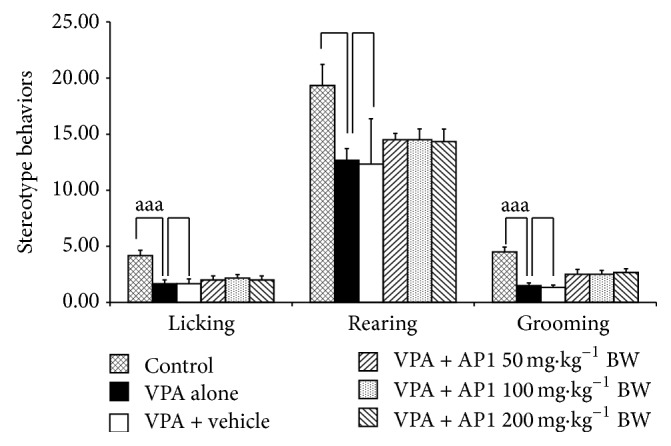
Effect of AP1 on stereotype behaviors, including rearing, grooming, and licking. Data are presented as mean ± SEM. ^aaa^ *p* value < 0.001, compared to the control group.

The effect of AP1 on stereotype behaviors was demonstrated in Figure 8. VPA significantly increased licking and grooming but produced no changes in rearing (*p* value < 0.001 all, compared to the control rats). In VPA-treated rats, neither the vehicle nor any of the doses of AP1 produced any significant changes in this parameter.

### 3.9. Effect of AP1 on Oxidative Stress Markers

The effect of AP1 on oxidative stress markers in the cerebellum is shown in Table 2. VPA-treated rats that also received the vehicle did not exhibit any significant changes in the superoxide dismutase (SOD), catalase (CAT), and glutathione peroxidase (GSH-Px) activities or in the malondialdehyde (MDA) level. AP1 at doses of 50, 100, and 200 mg·kg^−1^ BW significantly decreased the MDA level (*p* value < 0.05, 0.01, and 0.001, resp., compared to the VPA-treated rats that received the vehicle). AP1 at a dose of 100 mg·kg^−1^ BW was found to enhance the CAT activity (*p* value < 0.001, compared to VPA-treated rats that received the vehicle), whereas AP1 at a dose of 200 mg·kg^−1^ BW produced significant changes in both the CAT and the GSH-Px activities (*p* value < 0.01 and 0.05, resp., compared to VPA-treated rats that received the vehicle). No other changes were observed.

### 3.10. Effect of AP1 on the Cerebellum

**Figure 9:**
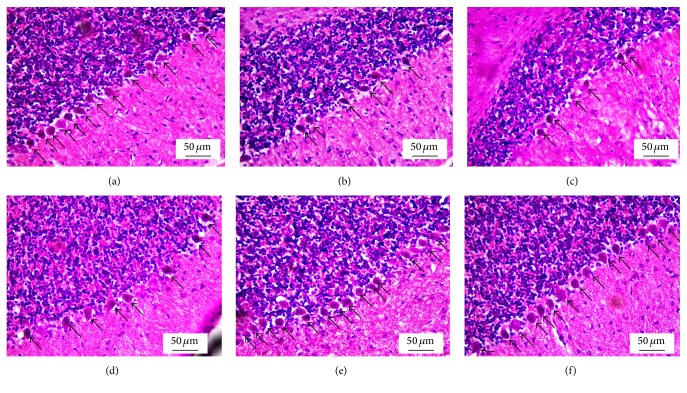
Effect of AP1 on the histomorphology of the cerebellum: (a) control group, (b) VPA-treated group, (c) VPA + vehicle group, (d) VPA + AP1 50 mg·kg^−1^ BW group, (e) VPA + AP1 100 mg·kg^−1^ BW group, and (f) VPA + AP1 200 mg·kg^−1^ BW group.

**Figure 10:**
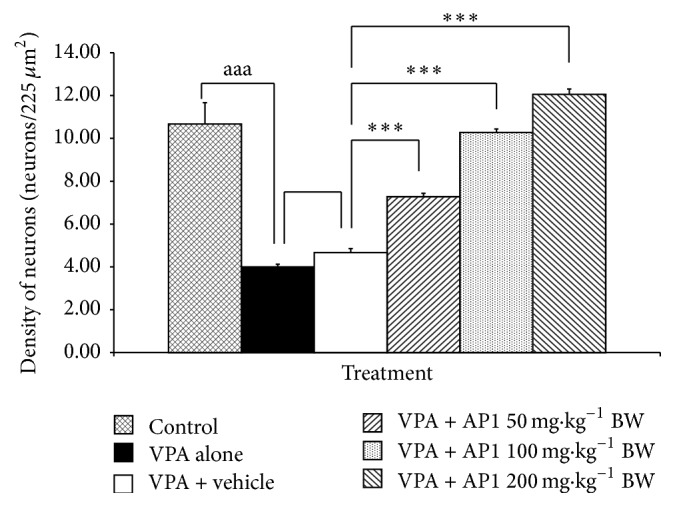
Effect of AP1 on the density of Purkinje cells in the left cerebellar hemisphere. Data are presented as mean ± SEM. ^aaa^ *p* value < 0.001, compared to the control group. ^*∗∗∗*^ *p* value < 0.001, compared to the VPA-treated group that received the vehicle.

The effects of AP1 on the cerebellum are shown in Figures 9 and 10.  Figure 9 showed that VPA significantly decreased both the numbers and the sizes of Purkinje cells in the cerebellum. No significant difference in these parameters was observed in VPA-treated rats that received the vehicle. However, all doses of AP1 significantly increased the sizes (Figure 9) and the densities of Purkinje cells (Figures 9 and 10) in the VPA-treated rats.

## 4. Discussion

In this study we have determined the density of Purkinje in left cerebellar hemisphere. It has been reported that cerebellar hemisphere is associated with motor function including movement, gait, and posture [35]. Recently, it has been reported to involve the cognitive function. This area works in cooperation with prefrontal cortex, posterior parietal cortex, and cortical motor area via the reciprocal connection [36]. The damage of cerebellum can disturb the functions involved with the connected areas mentioned earlier, resulting in the disorders of motor, sensory, and memory functions. In addition, the dysfunction of cerebellum also disturbed the function of prefrontal cortex, an area playing an important role on social behavior [37]. Since oxidative stress had been previously reported to play the crucial role on the neurodegeneration during development [38], we did suggest that the impairment of motor, sensory, learning, and memory and social behavior in VPA rats presented in this study might be due to the increased oxidative stress [38] leading to the degeneration of Purkinje cells in cerebellum which in turn disturbed the corticocerebellar circuit. Recent finding also demonstrates that cerebellum also plays an important role in emotion regulation and impairment of cerebellum can produce many psychotic disorders including anxiety [39]. Therefore, the anxiety behaviors in VPA rats might also occur as a result of the degeneration of Purkinje cells via the elevation of oxidative stress mentioned earlier. AP1 at all doses used in this study could improve Purkinje cell loss and autistic-like behaviors assessed in this study, except pain induced by thermal stimuli. The possible explanation for this phenomenon might be that the location of pain interpretation occurs at thalamus level and corticocerebellar loop plays smaller role on this sensation. However, further researches are required to provide the precise understanding about the mechanism.

It has been reported that lysine, the main amino acid presented in AP1, plays an important role in brain plasticity [40]. Therefore, the increased Purkinje cell in cerebellum might be associated partly with lysine content in AP1 which in turn increased the survival of Purkinje cell in cerebellum resulting in the improved autism-like behavior. In addition to lysine, abundance of tyrosine amino acid was also observed in AP1. Based on the previous information that tyrosine was an important precursor of dopamine and norepinephrine [41], the level of norepinephrine and dopamine in cerebellum might be increased leading to the increased long term depression which in turn enhanced learning and memory [42]. In addition to lysine and tyrosine, AP1 also contained anthocyanin, a substance possessing potent antioxidant effect [43]. Therefore, the decreased oxidative stress in this study might be associated with anthocyanin in AP1 which in turn gave rise to the increased Purkinje cells in VPA rats.

Taken all together, anthocyanin in AP1 might decrease oxidative stress in cerebellum, resulting in the enhanced Purkinje cell in cerebellum. The increased Purkinje cell in cerebellum might also be attributed to lysine as mentioned earlier. In addition, lysine and tyrosine in AP1 might also increase the activity of Purkinje cell giving rise to the increased long term depression (LTD) which in turn increased learning and memory. The increased Purkinje cells could also increase the function of corticocerebellar loop and the improved autism-like behavior. However, further researches are necessary to confirm the role of the main amino acids, lysine and tyrosine, and anthocyanin of AP1. In addition, the effect of gender difference should also be investigated.

## 5. Conclusion

AP1, the combined extract of purple rice and silkworm pupae, is the potential candidate to improve autism-like behavior in VPA model of autism. The possible underlying mechanism might occur via the decreased oxidative stress which in turn enhanced Purkinje cells in cerebellum and improved the cerebellum-related functions.

## Figures and Tables

**Table 1:** The contents of amino acids in AP1.

Amino acid	Concentration (mg/100 g)

Alanine	286.00
Arginine	<5.00
Aspartic acid	232.05
Cystine	192.36
Glutamic acid	943.45
Glycine	294.02
Histidine	1941.00
Hydroxylysine	<5.00
Hydroxyproline	56.50
Isoleucine	688.87
Leucine	955.43
Lysine	2012.00
Methionine	241.88
Phenylalanine	1019.00
Proline	289.02
Serine	274.25
Threonine	74.36
Tryptophan	104.14
Tyrosine	2153.00
Valine	561.69

**Table 2:** Effect of AP1 on the oxidative stress markers, including the activities of catalase (CAT), superoxide dismutase (SOD), glutathione peroxidase (GSH-Px), and the level of malondialdehyde (MDA). Data are presented as mean ± SEM. ^a^ *p* value < 0.05, compared to the control group. ^aaa^ *p* value < 0.001, compared to the control group. ^*∗*^ *p* value < 0.05, ^*∗∗*^ *p* value < 0.005, and ^*∗∗∗*^ *p* value < 0.001, compared to the VPA-treated group that received the vehicle.

Treatment	Cerebellum			
	CAT (units/mg protein)	SOD (units/mg protein)	GSH-Px (units/mg protein)	MDA (nmol/mg protein)
Control	0.53 ± 0.03	6.78 ± 0.36	15.39 ± 1.42	0.01 ± 0.02

VPA alone	0.28 ± 0.01^aaa^	2.49 ± 0.27^aaa^	8.42 ± 1.01^a^	0.07 ± 0.01^aaa^

VPA + vehicle	0.30 ± 0.03^aaa^	2.46 ± 0.32^aaa^	8.60 ± 1.50^a^	0.07 ± 0.01^aaa^

VPA + AP1 50 mg·kg^−1^ BW	0.32 ± 0.02	3.67 ± 0.57^*∗∗*^	10.29 ± 2.93	0.04 ± 0.01^*∗*^

VPA + AP1 100 mg·kg^−1^ BW	0.50 ± 0.04^*∗∗∗*^	6.65 ± 0.49^*∗∗*^	14.91 ± 2.06^*∗*^	0.02 ± 0.01^*∗∗*^

VPA + AP1 200 mg·kg^−1^ BW	0.46 ± 0.02^*∗∗*^	8.50 ± 1.8^*∗∗∗*^	15.82 ± 1.37^*∗*^	0.01 ± 0.00^*∗∗∗*^
